# Progressive Back Pain due to *Aspergillus nidulans* Vertebral Osteomyelitis in an Immunocompetent Patient: Surgical and Antifungal Management

**DOI:** 10.1155/2019/4268468

**Published:** 2019-07-02

**Authors:** Mark K. Lyons, Matthew T. Neal, Naresh P. Patel, Holenarasipur R. Vikram

**Affiliations:** Department of Neurological Surgery, Mayo Clinic Arizona, 5777 East Mayo Boulevard, Phoenix, AZ 85054, USA

## Abstract

**Case Report:**

*Aspergillus* osteomyelitis is a destructive and progressive infection that has been described both in immunosuppressed and in immunocompetent hosts. We describe a case of lumbar vertebral osteomyelitis in a 61-year-old immunocompetent patient due to *Aspergillus nidulans* that was successfully treated with a combination of extensive surgical debridement, spinal stabilization, and a prolonged course of antifungal therapy. Imaging demonstrated findings consistent with L3 discitis. The biopsy grew *Aspergillus* fungus and was treated with vorconizole. Imaging showed progressive destructive osteomyelitis at L3-L4. Patient underwent anterior L3 and L4 partial corpectomies, anterior interbody fusion L3-L5, and posterior T11-S2 pedicle screw and rod fixation. Antifungal treatment resulted in resolution of infection. *Aspergillus* markers remain negative. One year following definitive treatment, the patient's back pain remains resolved.

**Conclusion:**

Definitive surgical resection of the infection, spinal stabilization, and aggressive antifungal therapy were required to eradicate the infection.

## 1. Introduction

*Aspergillus* osteomyelitis is an uncommon extra pulmonary manifestation of invasive aspergillosis [1, 2]. Although *Aspergillus* osteomyelitis is rare, the vertebral bodies are the most commonly infected site followed by spondylodiscitis [1, 3, 4]. Immunocompromised hosts are more susceptible to invasive aspergillosis. They include solid organ recipients; severe and sustained neutropenia in patients with hematologic malignancies, following hematopoietic stem cell transplantation; and those receiving high-dose steroids. Other risk factors include poorly controlled diabetes mellitus, chronic obstructive pulmonary disease, and intravenous drug users [1, 3–7]. The presenting symptoms and imaging characteristics of fungal osteomyelitis are nonspecific with back pain being the predominant complaint. In immunocompetent hosts, either hematogenous infection or a prior spinal procedure has been implicated as the cause of aspergillosis osteomyelitis [4]. The excellent review by Gamaletsou et al. looking at the various reported cases of aspergillosis osteomyelitis, where the subtype of the fungus was identified, found only three cases of *Aspergillus nidulans* osteomyelitis in the literature search of aspergillosis spinal infections [1]. The most common subtype was *Aspergillus fumigatus* by far. The majority of patients with *Aspergillus* osteomyelitis require surgical debridement and antifungal treatment. The reported fatality rate in patients with *Aspergillus* osteomyelitis was 23% in 86 patients culled from the reported literature [1]. This case highlights the importance of adequate tissue sampling for culture, histopathology, and noninvasive fungal studies to establish a timely etiologic diagnosis that leads to correct diagnosis and aggressive surgical and prolonged antifungal therapy with resolution of infection.

## 2. Case Report

**Figure 1:**
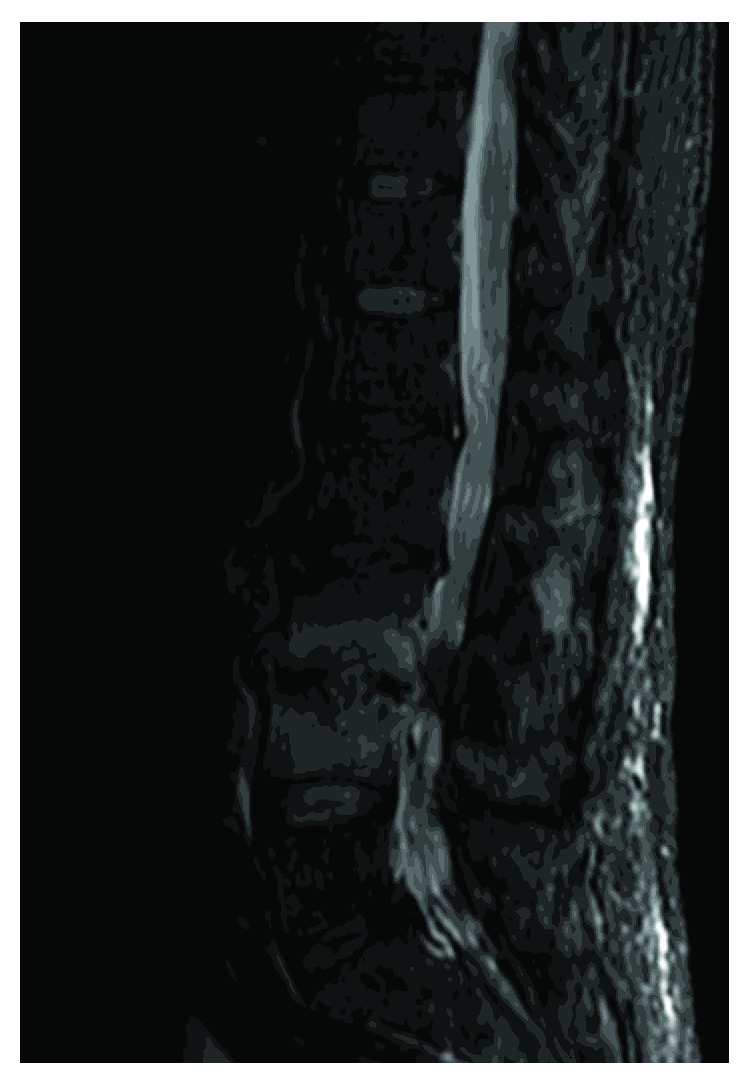
Sagittal STIR MR demonstrating signal abnormality in the L3 disc space with endplate irregularity.

A 61-year-old male in good health with a history of mild low back pain over several years began developing subacute onset of worsening of back pain and lower extremity pain over six weeks. He had a lumbar steroid injection 18 months prior. He was initially evaluated at an outside institution where a magnetic resonance (MR) imaging study of the spine was obtained which was interpreted as normal. Due to unremitting back pain, he presented to our institution where a repeat MR imaging study was obtained demonstrating abnormal intradiscal signal between the L3 and L4 vertebral bodies consistent with disc space infection and adjacent vertebral osteomyelitis (Figure 1). In addition, a small amount of enhancing phlegmon was noted. The patient underwent a CT-guided biopsy of the disc space which was nondiagnostic with negative cultures. At the time of his initial presentation, his white count was 7.2 with C-reactive protein of 84 and a sedimentation rate of 84. Blood cultures were obtained and were negative. The patient was initially placed on broad-spectrum antibiotics. With ongoing symptoms and no identifiable pathogen, an open L3 disc space biopsy was performed three days later. Fungal culture revealed growth of *Aspergillus nidulans*. Susceptibility testing was performed. Serum *Aspergillus* antigen and Fungitell assays were significantly elevated. He was started on voriconazole 200 mg orally twice per day with excellent serum levels.

**Figure 2:**
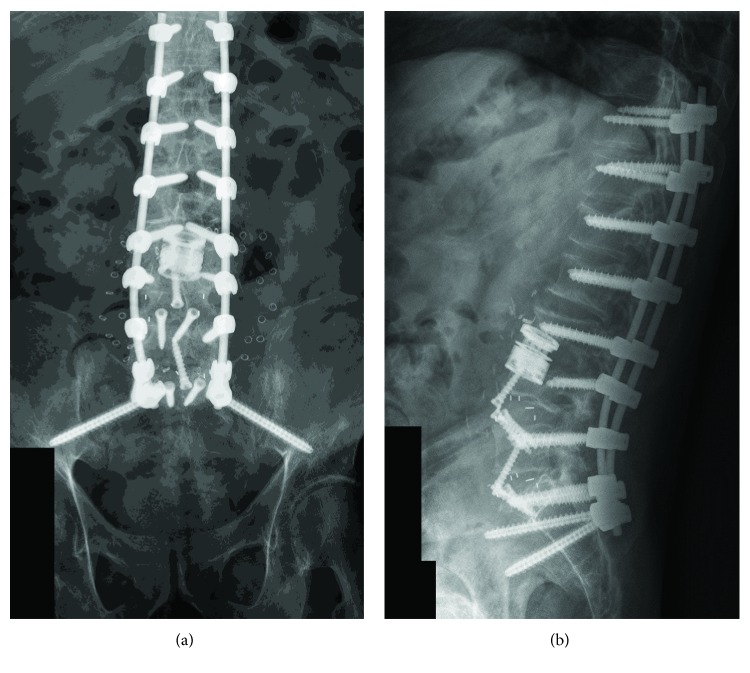
(a, b) Standing anterior-posterior and lateral X-rays demonstrating T11-S2 bilateral pedicle screw and rod fixation with bilateral iliac screws. Anterior interbody L4 and L5 PEEK graft. Anterior L3 intradiscal expandable cage and bone graft.

Initially, the patient's back pain began to improve. However, 4 weeks after the disc space biopsy and initiation of antifungal treatment, his symptoms rapidly progressed. The patient's laboratory studies revealed a sedimentation rate greater than 100 and his C-reactive protein was elevated at 25. His *Aspergillus* antigen was 3.75 which was a slight increase from his level of 3.37 about 4 weeks earlier. In addition, his voriconazole level was 0.9 which was down from 4.1 3 weeks prior. A follow-up MR demonstrated persistent T1 and STIR sequence signal abnormalities involving the L3 and L4 vertebral bodies. There was progressive bony destruction of the L3 and L4 vertebral bodies as well as focal progressive kyphosis. In addition, new wedge compression deformities of L1, L2, and L5 had developed in the interim. Presumably these were related to poor bone density. Although the patient had only undergone six weeks of antifungal therapy, the progression of symptoms, laboratory markers, and radiographic deterioration prompted recommendation for surgical intervention. The significant risk of mortality with *Aspergillus nidulans* spinal osteomyelitis treatment was the key factor in the recommendation for surgical debridement [1–8]. Treatment recommendations for spinal osteomyelitis and suspected treatment failure may be guided by unchanged or increasing inflammatory markers after four weeks of treatment [9, 10]. Infectious Disease Society of America recommendations in cases of suspected treatment failure include follow-up MR imaging and additional tissue samples [9]. In cases of radiographic evidence of treatment failure, surgical debridement should be considered [9]. Given that all of these findings were present in our patient, aggressive surgical debridement and spinal stabilization was advised. The patient underwent an anterior lumbar discectomy at L3-L4 with partial corpectomies of the adjacent L3 and L4 vertebral bodies. After complete debridement of the infected disk and adjacent bone, an expandable titanium corpectomy cage was used for reconstruction. It was noted that the L3 disc material was very fibrotic, and intraoperative microbiology testing confirmed fungal hyphae consistent with aspergillosis. Anterior lumbar fusion procedures were also performed at L4-5 and L5-S1 using lordotic polyetheretherketone cages containing integrated screws. The anterior fusion construct corrected lumbar lordosis-pelvic incidence mismatch, stabilized the anterior and middle spinal columns, and provided a favorable arthrodesis environment. Circumferential spinal fixation was also desired due to the patient's poor bone quality and placement of the expandable cage (Figures 2(a) and 2(b)). The patient was repositioned and then underwent a posterior bilateral pedicle screw and rod fixation from T11-S2 with iliac screws. We recommended a multilevel anterior and posterior fusion in order to maximize the chance of successful arthrodesis and reduce the chance of the need for further stabilization in the setting of this potentially fatal disease. The patient tolerated the surgical procedure well and reported immediate improvement in his back and leg pain. After surgery, he was initiated on voriconazole 300 mg twice daily along with caspofungin 50 mg daily for 6 weeks.

After 6 weeks, caspofungin was stopped and he received oral voriconazole at 300 mg twice daily for 7 months with adequate serum levels. Voriconazole was subsequently discontinued after the patient developed severe photosensitivity, and posaconazole 300 mg per day was initiated. Follow-up MR studies demonstrated resolution of the signal abnormality at the L3-L4 level without evidence of residual or recurrent infection. There was no evidence of spinal canal compromise. Due to the severity of the patient's pain on presentation, the patient could not tolerate standing X-rays preoperatively. However, standing spine X-rays at one year confirms good sagittal balance. The study demonstrated T11-S1 posterior fusion with pedicle screws and posterior stabilization rods with bilateral iliac screws, anterior discectomy with interbody grafts at L4-5 and L5-S1, and partial L3 and L4 corpectomies with an expandable cage. Imaging at two and three years is planned for deformity correction assessment. Serum *Aspergillus* antigen and Fungitell assay trended down and have normalized. His sedimentation rate and C-reactive protein levels are also normal. His back pain has significantly improved without any residual neurologic defects. Since he is now a year out from his extensive surgery without any evidence of persistent or recurrent infection, the plan is to discontinue posaconazole and monitor him closely.

## 3. Discussion

*Aspergillosis* osteomyelitis is an uncommon condition, and *Aspergillus nidulans* is a rare species of the fungus reported in the literature. In the limited number of vertebral aspergillosis cases reported in the literature, the complete response rate to treatment is approximately 50% of patients [1]. Chronic granulomatous disease is the most common immunodeficiency-related condition associated with *Aspergillus* osteomyelitis, especially in the pediatric population [11]. It is generally believed that the mechanism of infection is hematologic spread or direct inoculation, particularly in the immunocompromised population. However, as in our patient, nonimmunocompromised patients are also a population at risk for *Aspergillus* osteomyelitis [4, 12]. Studemeister and Stevens reported on a literature review of *Aspergillus* vertebral osteomyelitis in immunocompetent hosts [4]. The most common presenting symptom was back pain with *Aspergillus fumigatus* being the most common subtype identified. They identified 21 cases with the majority acquired by hematogenous spread; however, only 25% of those cases occurred after a spinal operation [4]. The mortality rate was 20% in those combined cases. The vertebral bodies are the most common infected site in *Aspergillus* osteomyelitis. The route of infection can occur by transpleural extension into adjacent vertebral bodies, by direct inoculation, and by hematogenous spread. And in our patient, given that the infection was at the L3 interspace, it is more likely that this was a hematogenous spread of the organism.

Inflammatory markers are often elevated in patients with *Aspergillus* osteomyelitis. However, these are nonspecific markers and underscored the importance of serum *Aspergillus* antigen testing in patients considered at risk for this disease. The management of *Aspergillus* osteomyelitis includes surgical debridement and antifungal therapies. The report by Gamaletsou et al. on the epidemiology and management of *Aspergillus* osteomyelitis supports this conclusion [1]. There is a significant reduction in the reported frequency of relapse in patients undergoing surgical debridement [1, 3, 12, 13]. The relatively low number of patients prevents categorizing risk by fungal subtype. It appears that the overall response rates to the various antifungal agents are similar. As in our case, the patient required aggressive surgical debridement, spinal stabilization, and a course of prolonged antifungal therapy in order to successfully treat his infection. The optimal duration of antifungal therapy is not known in *Aspergillus* osteomyelitis. The duration of treatment needs to be based on the potential cumulative nephrotoxicity as well as the morbidity of prolonged therapy in aspergillosis osteomyelitis [14]. However, our patient required discontinuation of the voriconazole secondary to severe photosensitivity side effect. He was able to start on posaconazole and has tolerated that medication well. Although the number of cases of *Aspergillus* osteomyelitis treated with posaconazole is limited, the available information suggests similar clinical response [15].

*Aspergillus* osteomyelitis is a severe infection that if not aggressively treated can potentially be fatal [1, 3, 4]. The relapse rate does appear to be higher without aggressive surgical intervention despite adequate and appropriate antifungal therapies. When clinical and anatomical considerations allowed for safe surgical intervention, it appears that aggressive surgical debridement and appropriate spinal stabilization followed by antifungal therapy may offer the best chance for the patient to have a good clinical outcome.
